# Mitigating underreported error in food frequency questionnaire data using a supervised machine learning method and error adjustment algorithm

**DOI:** 10.1186/s12911-023-02262-9

**Published:** 2023-09-09

**Authors:** Anjolaoluwa Ayomide Popoola, Jennifer Koren Frediani, Terryl Johnson Hartman, Kamran Paynabar

**Affiliations:** 1 Georgia Institute of Technology, Atlanta, GA USA; 2 Nell Hodgson Woodruff School of Nursing, Emory University, Atlanta, GA USA; 3 Rollins School of Public Health, Emory University, Atlanta, GA USA

**Keywords:** Food frequency questionnaire, Machine learning, Supervised learning, Underreporting, Measurement error, Error adjustment model

## Abstract

**Background:**

Food frequency questionnaires (FFQs) are one of the most useful tools for studying and understanding diet-disease relationships. However, because FFQs are self-reported data, they are susceptible to response bias, social desirability bias, and misclassification. Currently, several methods have been created to combat these issues by modelling the measurement error in diet-disease relationships.

**Method:**

In this paper, a novel machine learning method is proposed to adjust for measurement error found in misreported data by using a random forest (RF) classifier to label the responses in the FFQ based on the input dataset and creating an algorithm that adjusts the measurement error. We demonstrate this method by addressing underreporting in selected FFQ responses.

**Result:**

According to the results, we have high model accuracies ranging from 78% to 92% in participant collected data and 88% in simulated data.

**Conclusion:**

This shows that our proposed method of using a RF classifier and an error adjustment algorithm is efficient to correct most of the underreported entries in the FFQ dataset and could be used independent of diet-disease models. This could help nutrition researchers and other experts to use dietary data estimated by FFQs with less measurement error and create models from the data with minimal noise.

**Supplementary Information:**

The online version contains supplementary material available at 10.1186/s12911-023-02262-9.

## Introduction

Food frequency questionnaires (FFQ) are often used in large prospective cohort studies to assess habitual dietary intake and understand diet-disease relationships [1]. These questionnaires are faster to administer and take less resources to analyze in a large cohort compared to multiple 24-h dietary recalls (24HR) or multi-day dietary food records (FR). Dietary assessment that utilizes 24HR may reduce measurement error; however, archetypal cohorts and some more recent studies use FFQs to measure dietary patterns. Cohorts, such as Reasons for Geographic and Racial Differences in Stroke (REGARDS) and Atherosclerosis Risk in Communities Study (ARIC), contain older versions of FFQs. With new technologies emerging, such as omics, clinical samples from these studies are driving new research questions that would benefit from including dietary information. For example, banked samples from the ARIC study have been used for metabolomics analyses [2]. These types of analyses would benefit from the comparison between habitual dietary patterns gleaned from the available FFQs and omics data.

Historically, dietary assessment has known limitations. Self-reported data of any kind, but especially dietary assessment data, introduces recall bias, response bias, social desirability bias, and misclassification [3–6]. These ultimately render the dataset inefficient for any future predictions or studies, hereby limiting the range of new findings that can be drawn from these studies. Therefore, it is crucial to combat the measurement error challenges in these datasets for optimal usability.

Currently, several methods have been used to adjust for measurement error. One of the most common methods is regression calibration, in which the conditional expectation of the true long-term intake of the variable replaces the FFQ intake given a vector of error-free covariates [7–9]. This supports the assumption that there is underlying truth in the dataset. However, this method has some limitations. It relies heavily on the use of other tools such as 24HR which could introduce additional bias into the model. The solution is to find the most efficient way to use the FFQ dataset without relying on internal calibration.

Participants, particularly those with a health condition, sometimes underreport or overreport certain types of food for a variety of reasons [10–13]. We apply the assumption of underlying truth in each dataset, which could be determined from both the healthier participants and the known reasons for under or overreporting [10–16]. For demonstration purposes, we obtained a dataset of university employees that were considered relatively healthy, with either no disease or well controlled disease. Each participant was asked to complete an FFQ at every study visit for the duration of the multi-year study. We used this dataset to build a predictive model to correct over and underreported responses in a full semi-quantitative food frequency questionnaire.

Our objective was to reclassify misreported foods to adjust for known measurement error. We proposed a supervised machine learning approach which uses a random forest classifier to label the responses in the FFQ based on the input dataset. In addition, an algorithm was written based on the newly predicted class probabilities derived from the random forest model.

## Material and methods

### FFQ data participants

This work is based on information from the Emory Predictive Health institute and Center for Health Discovery and Well Being Database (CHDWB) which has been described previously [17]. Briefly, the CHDWB cohort at Emory University in Atlanta, Georgia, USA, was an observational study designed to investigate the effects of clinical self-knowledge and health partner counseling on various health outcomes. In the present study, we included 819 participants for which complete FFQ data at various time points was available. Individuals with poorly controlled chronic disease or acute illness were excluded. Demographic information and potential covariates (e.g., body mass index and personal health history) were collected from the CHDWB cohort database. The FFQ was the Block 2005 [18] delivered in an electronic format. This questionnaire was filled out by the participant prior to study visits via an online portal. These were not verified by the study staff prior to summary calculations conducted by the developer (Nutritionquest, Berkeley, CA, USA). It is assumed that some entries are either underreported or overreported.

Blood draws were performed in a fasting state and blood lipids and blood glucose were measured by commercially available assays (Quest Diagnostics, Madison, NJ, USA). Body fat percentage was determined using dual x-ray absorptiometry (Lunar iDXA, General Electric, Chicago, IL, USA). Weight was measured in athletic clothing without shoes on a research grade scale (Tanita, Tokyo, Japan) and height was measured using a standard stadiometer. BMI was calculated using kg of body weight divided by height in meters squared.

### Exploratory data analysis

Initial data analysis included missing data assessment and correlation analysis. The CHDWB dataset contained demographics, clinical biomarkers, and FFQ data reflecting habitual diet in the past year. The original dataset contained 593 variables and 3193 unique samples, including missing data points. Heatmaps were used to visualize the correlations between food frequency and demographic information. Due to high correlations between variables, it was fair to assume a low rank data assumption, which allowed the underlying ground truth to be determined from the present data to infer accuracy.

### Variable selection

**Fig. 1:**
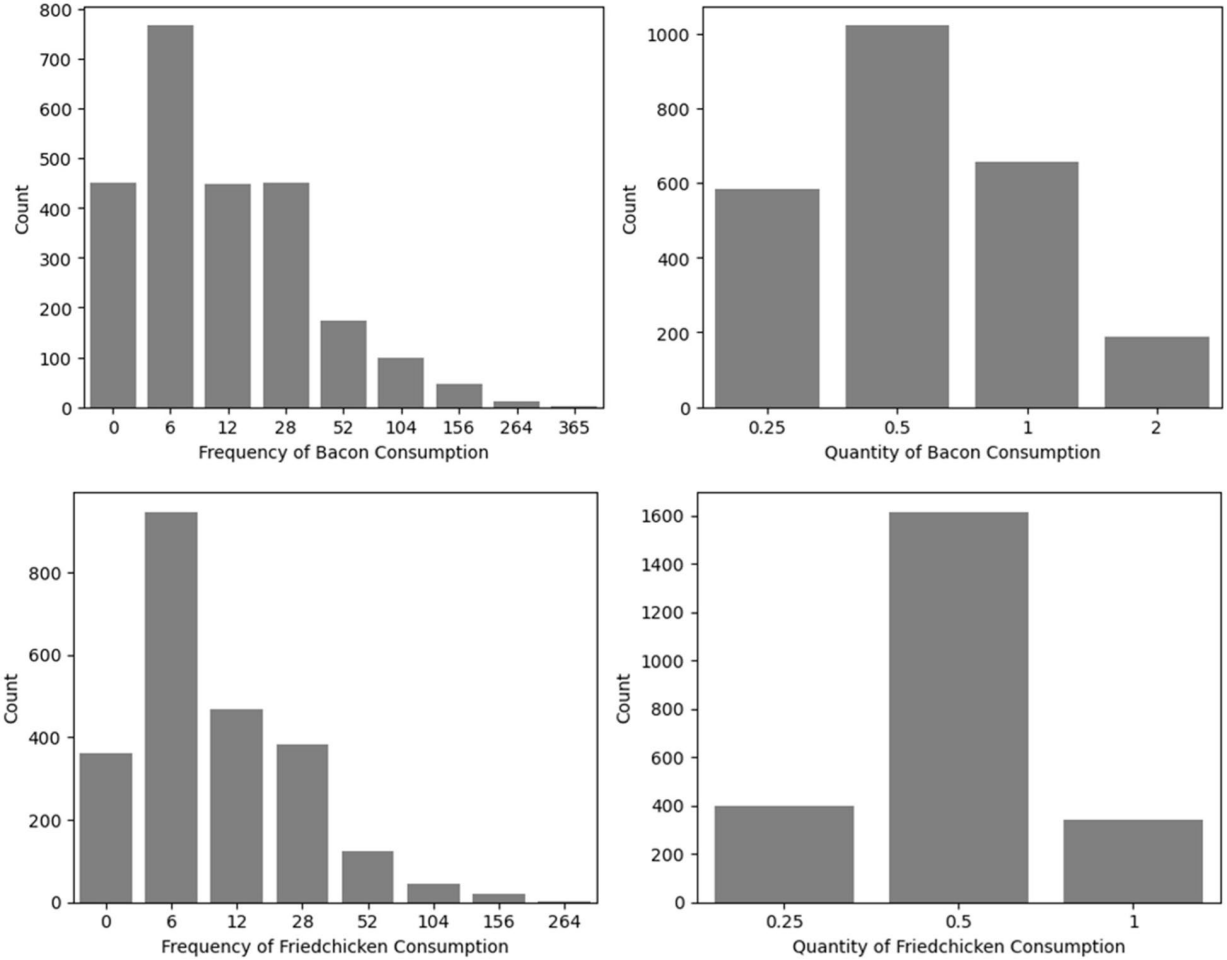
Bar plots showing bacon and fried chicken frequency (F) and quantity counts (Q). X-axis represents the consumption frequency(F) per year and quantity (Q) in cups, and Y-axis represents the frequency of participants (count) distributed among the respective categories

Underestimation errors in the FFQ are the most common issues [19]; thus, we chose to focus our analyses on this problem. Variables were selected based on fat content as those foods are typically underreported [20]. The four selected variables used as individual responses are the frequency and quantity of bacon consumed and the frequency and quantity of fried chicken consumed. The frequency count of the values of these variables can be seen in Fig. 1

These selected variables are ordinal. As mentioned above, these were used as responses in four different classification models where accurate responses were predicted.

We chose the following as explanatory variables: blood levels of low-density lipoprotein (LDL), total cholesterol, and glucose, body fat percentage, and body mass index (BMI) [1]. The explanatory variables selected for responses were chosen based on the assumption that they would have low measurement error because of their objective nature. These explanatory variables have proven relationships with frequency and quantity of bacon and fried chicken [14–16]. Age and sex, which are generally reported accurately, were added as demographic explanatory variables.

### Training machine learning-based error adjustment model

The proposed error mitigation approach relies on the premise that some groups of participants may be more likely to report their food consumption more accurately, while others tend to underreport/overreport their unhealthy/healthy food consumption. Another assumption made in this study is that some of the objectively measured variables including LDL cholesterol, total cholesterol, blood glucose, body fat percentage and anthropometric measures, and participant characteristics, including age and sex, are correlated with food consumption habits. For example, participants that have a high saturated fat diet may have high blood cholesterol concentrations [14–16].

The overview of the proposed framework is given in Fig. 3. We first split the dataset into two groups representing healthy and unhealthy participants. The healthy group data were defined by using certain cutoffs for the body fat percentage, age and sex which classified participants by their health risks (for the specific health risk classification table, please refer to Tables A2.1 and A2.2). While the participants with excellent, good and normal health risks have their responses defined as the healthy samples of the data consisting of 384 responses and 9 variables, the rest are defined as the unhealthy group data- consisting of 2238 responses and 9 variables. Then, based on the foregoing assumptions, we used the healthy group data to train a predictive model that quantifies the relationship between lab test variables and participant characteristics within the food frequency variables. Specifically, since the FFQ data are categorical we use random forest (RF) classification to build the predictive model. Using cross-validation, we tuned the hyperparameters and selected the tree depth that showed the best model performance and highest training accuracy [21]. RF was selected over logistic regression due to higher performance, higher capability of capturing nonlinear relationships, robustness to overfitting, and ability to rank the importance of predictors [21].

After this relationship was learned, the trained predictive model was used to predict the food frequency variables for the unhealthy group based on their lab test results, BMI, sex, and age. Finally, the predicted value was compared to the original value reported by the participants in the FFQ dataset in the unhealthy group data where the likelihood of underreporting is higher. If the original FFQ response is smaller by any amount than the predicted value, it will be replaced by its prediction. Otherwise, it is kept unchanged or modified according to the procedure described in “Applying error adjustment model” section.

### Applying error adjustment model

**Fig. 2:**
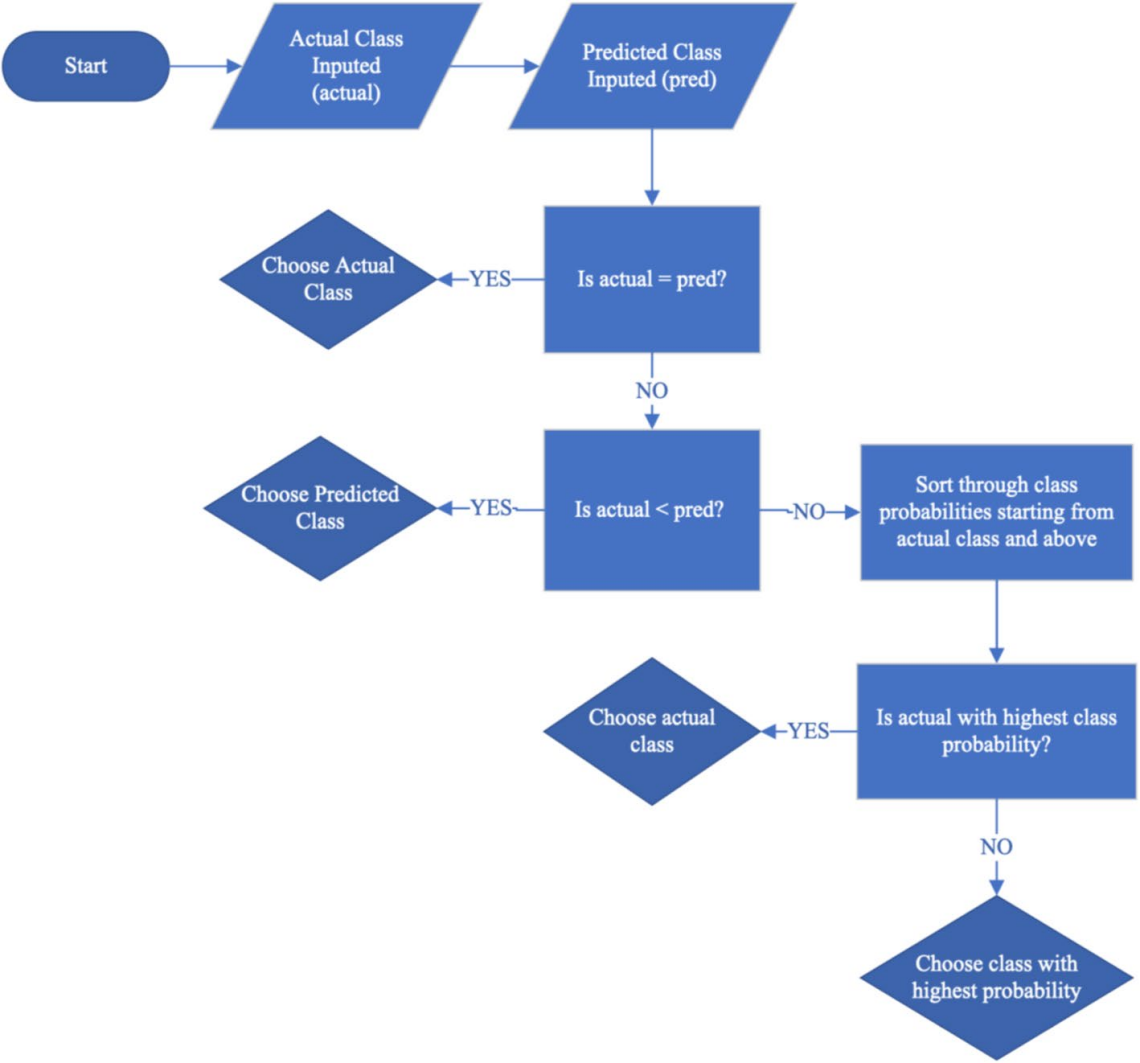
Flowchart showing the algorithm for error adjustment

In the final step of the proposed error mitigation approach, the trained RF prediction model used the objectively measured variables, anthropometric variable and participant characteristics to determine the FFQ response category with the highest likelihood. Additionally, the prediction model can provide the likelihood of other categories for each response. For a response there are *L* categories of ${C}_{\left(1\right)}, {C}_{\left(2\right)},\dots ,{C}_{\left(L\right)}$ that are sorted in descending order with respect to their corresponding probabilities ${P}_{\left(1\right)}, {P}_{\left(2\right)},\dots ,{P}_{\left(L\right)}$ obtained by the RF model. First, the class with the highest probability, i.e., ${C}_{\left(1\right)}$ is compared with the reported response in the FFQ dataset. For healthy food where the likelihood of overreporting is higher, the FFQ response is replaced with the category lower than the reported FFQ response that has the largest probability, i.e., ${C}_{\left(i\right)}$ where $i=\mathrm{argmax}\left\{{P}_{\left(i\right)}; i=\mathrm{1,2},\dots ,L\right\}; {C}_{\left(i\right)}<{C}_{R}$. For unhealthy food where the likelihood of underreporting is higher, the FFQ response is replaced with the category higher than the reported FFQ response that has the largest probability, i.e., ${C}_{\left(i\right)}$ where $i=\mathrm{argmax}\left\{{P}_{\left(i\right)}; i=\mathrm{1,2},\dots ,L\right\}; {C}_{\left(i\right)}>{C}_{R}$. A summary of this procedure is given in Fig. 2.

### Validation studies using simulation

The purpose of using a simulation study was to evaluate the performance of our proposed method. Unlike the FFQ dataset, the ground truth is known in simulated data. The main goal was to analyze how the proposed model would perform when the data simulated is very similar to real data, in this case FFQ data.

To simulate the dataset, we randomly generated a synthetic multinomial dataset using the make_classification function from Scikit-learn library. For simplicity, the synthetic dataset was meticulously engineered to emulate the characteristics observed in the FFQ data. For example, since the case study has 8 variables and 7 classes, the synthetic dataset was constructed to maintain those parameters, incorporating the 7 classes within its responses and 8 distinct variables across observations. In this study, we assumed the response represents consumption of unhealthy food (e.g., bacon frequency level). We followed this procedure to generate 1000 responses for 1000 simulated participants.

To ensure that our method is robust, we also tried two other simulation settings. The second setting involved generating another synthetic multinomial dataset with a smaller number of categories. We chose 4 classes which is similar to the bacon and fried chicken quantity levels. In the third setting, we generated a synthetic multinomial dataset with more distinct variables across observations and more responses, i.e., 15 variables and 10,000 responses.

The datasets were split into healthy and unhealthy subsets using the train and test split with a test ratio of 0.3 to mimic the process in the original food frequency dataset. This means 70% of each synthetic data was classified as the healthy subset and the rest were classified as the unhealthy subset. To induce underreported responses, responses from the unhealthy subset were randomly altered to lower categories such that 50% of responses decreased by one level, 20% decreased by two levels and finally, 10% decreased by three levels, and the rest remained the same.

**Fig. 3:**
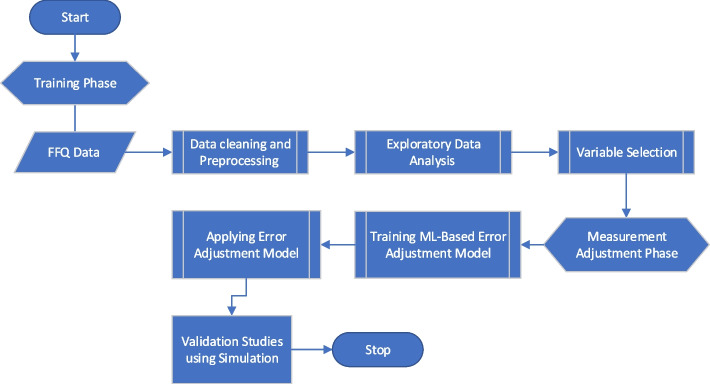
Flowchart describing the methods for machine learning (ML) error adjustment model

Next, following our proposed approach, we trained the error adjustment model using healthy group data and used the trained model to adjust the response for the unhealthy subset. Figure 3 depicts a summary of all the methods used.

## Results

### FFQ data results

**Fig. 4:**
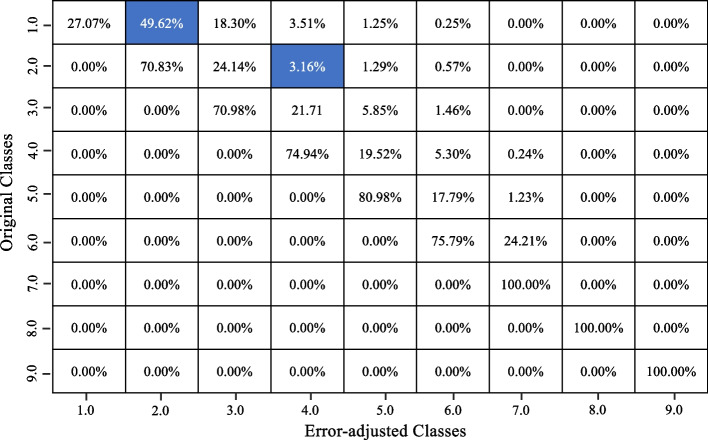
Confusion matrix showing the changes between the original and adjusted responses for bacon frequency

**Fig. 5:**
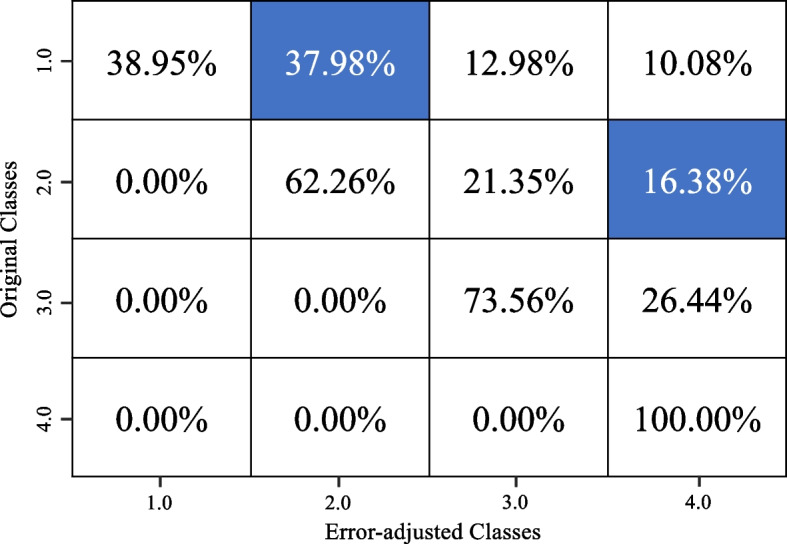
Confusion matrix showing the changes between the original and adjusted responses for bacon quantity

**Fig. 6:**
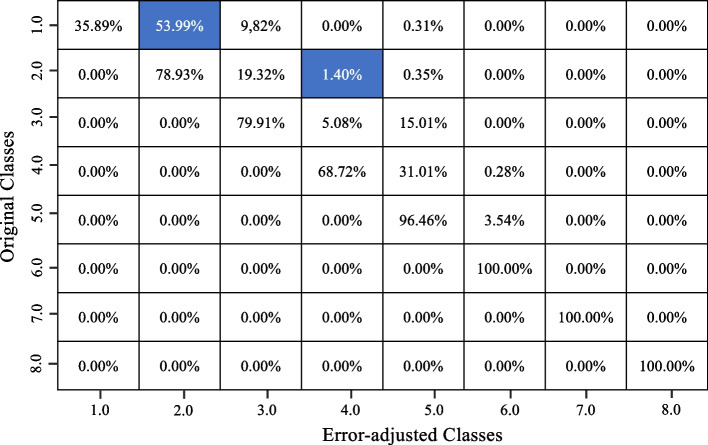
Confusion matrix showing the changes between the original and adjusted responses for fried chicken frequency

**Fig. 7:**
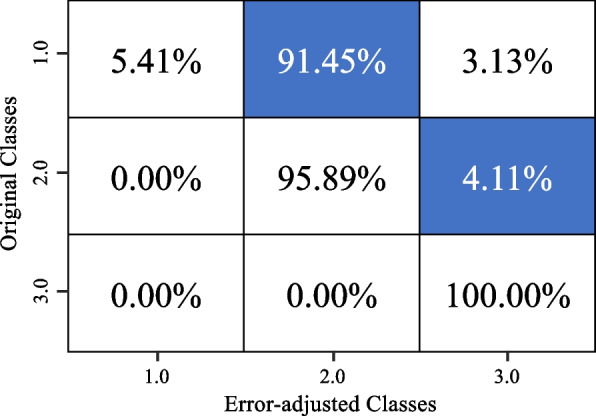
Confusion matrix showing the changes between the original and adjusted responses for fried chicken quantity

Four RF classifier models were built, and the underestimation algorithm was used to correct the initial underreported entries in the FFQ data for the frequency and quantity of bacon consumed and frequency and quantity of fried chicken consumed. Four confusion matrices comparing the initial entries of the dataset and the final corrected entries of bacon frequency, bacon quantity, fried chicken frequency and fried chicken quantity are shown in Figs. 4, 5, 6 and 7 respectively.

Looking at the frequency of bacon consumed, the RF classifier model has a model accuracy of 84.4%. This model demonstrates a precision score of 0.801, a recall score of 0.805 and ultimately resulting in an F1 score of 0.807. In the confusion matrix, we see that many of the entries stay the same; however, a couple of entries moved to higher classes. In Fig. 4, about 50% of ‘class 1’ entries became ‘class 2’, and 3% of ‘class 2’ entries became ‘class 4’. Looking at the quantity of bacon consumed, the RF classifier model has a model accuracy of 87%. This model demonstrates a precision score of 0.826, a recall score of 0.818 and ultimately resulting in an F1 score of 0.820 . In the confusion matrix (Fig. 5), we see that many of the entries stay the same, with some changes detected in classes above the initial class. For instance, about 38% of ‘class 1’ entries became ‘class 2’ and 16% of ‘class 2’ entries became ‘class 4’.

For the frequency of fried chicken consumed, the RF classifier model has a model accuracy of 91.6%. This model demonstrates a precision score of 0.882, a recall score of 0.861 and ultimately resulting in an F1 score of 0.858. In the confusion matrix, we see that many of the entries stay the same; however, a couple of entries moved to higher classes. In Fig. 6, 54% of ‘class 1’ entries became ‘class 2’, and 1.4% of ‘class 2’ entries became ‘class 4’. Looking at the quantity of fried chicken consumed, the RF classifier model has a model accuracy of 93.1%. This model demonstrates a precision score of 0.912, a recall score of 0.902 and ultimately resulting in an F1 score of 0.896. In the confusion matrix (Fig. 7), we see that many of the entries stay the same, with some changes detected in classes above the initial class. For instance, about 91% of ‘class 1’ entries became ‘class 2’ and 4% of ‘class 2’ entries became ‘class 3’.

### Simulation results

**Fig. 8:**
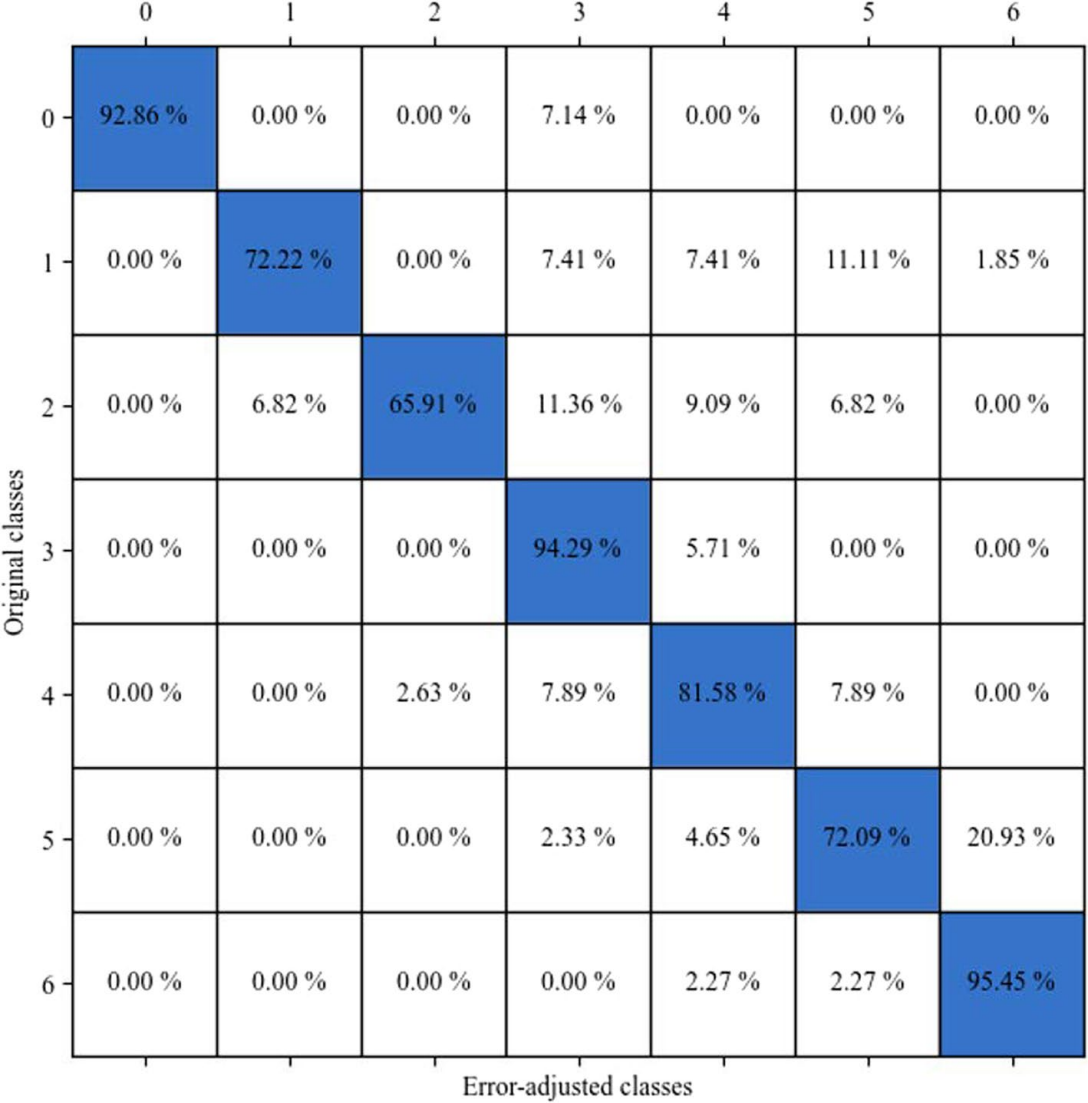
Confusion matrix showing the changes between the original simulated data response and the adjusted responses

From the simulation study, the RF classifier model has a model accuracy of 78.5%. This model demonstrates a precision score of 0.794, a recall score of 0.786 and ultimately resulting in an F1 score of 0.785. After applying the error adjustment algorithm, we saw that some of the entries in ‘class 1’ became ‘class 2’ entries. To ensure the proposed method worked using this simulated study, we compared the originally simulated data responses to the final simulated responses using another confusion matrix (Fig. 8). From this, we see that the underestimation algorithm accurately classified the classes with 82.06% average accuracy rate.

**Fig. 9:**
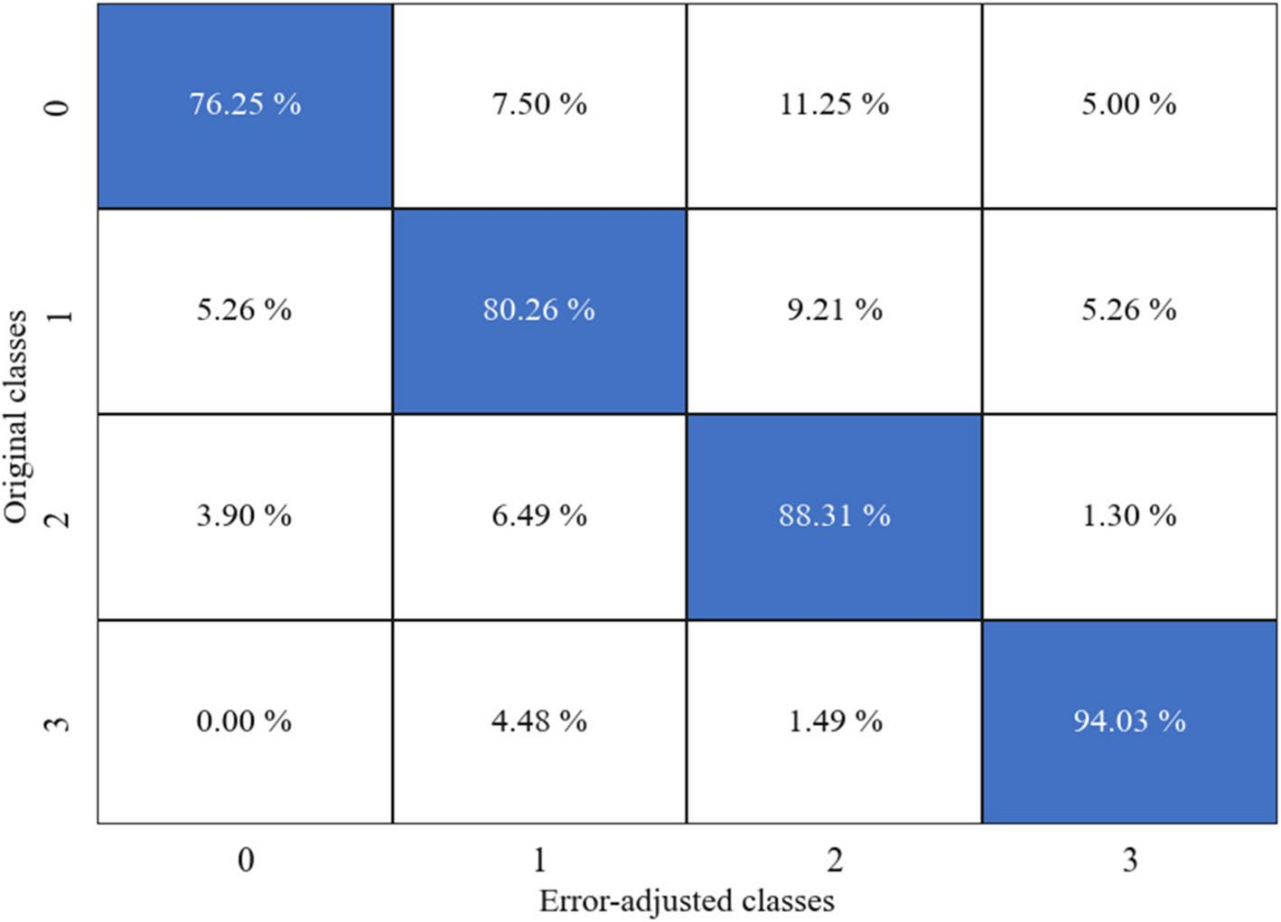
Confusion matrix showing the changes between the original simulated data response for the second setting and the adjusted responses

To ensure that our model is robust, the second setting resulted in a model accuracy of 90%. This model demonstrates a precision score of 0.901, a recall score of 0.900 and ultimately resulting in an F1 score of 0.90. After applying the error adjustment algorithm, we saw that some of the entries in ‘class 1’ became ‘class 2’ entries. Finally, we compared the originally simulated data responses to the final simulated responses using another confusion matrix (Fig. 9). From this, we see that the underestimation algorithm accurately classified the classes with 84.71% average accuracy rate.

**Fig. 10:**
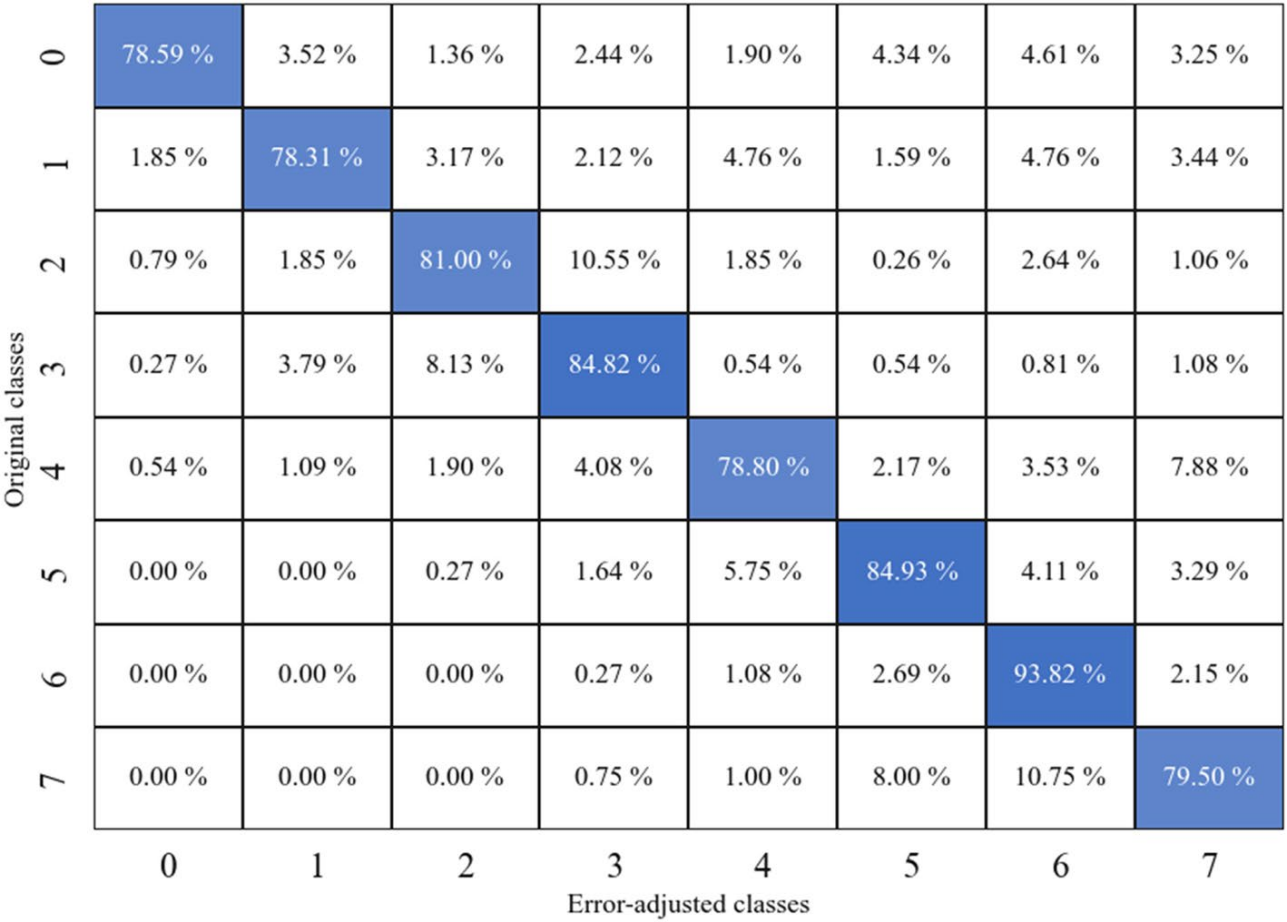
Confusion matrix showing the changes between the original simulated data response for the third setting and the adjusted responses

Furthermore, the third setting resulted in a model accuracy of 80.71%. This model demonstrates a precision score of 0.807, a recall score of 0.806 and ultimately resulting in an F1 score of 0.805. After applying the error adjustment algorithm, we saw that some a similar shift pattern in the entries. Finally, we compared the originally simulated data responses to the final simulated responses using another confusion matrix (Fig. 10). From this, we see that the underestimation algorithm accurately classified the classes with 82.47% average accuracy rate. These indicate that our proposed method worked as expected, since we knew the true original entries, and show that it is robust.

## Discussion

As seen from the results, we have high model accuracies ranging from 77.5% to 91.6% in participant collected data. This shows that our two-step method of using RF classifier and an error adjustment algorithm is efficient in correcting most of the underreported entries in the FFQ dataset. Looking at the confusion matrices of bacon and fried chicken frequency, we can see that misclassification due to underestimation is greatly reduced as the self-reported classes are moved to their “true” classes, with accuracies of 83.1% and 91.6%, respectively. The same can be seen in the bacon and fried chicken quantity variables as the misclassified observations are adjusted and moved to their true classes with accuracies of 77.5% and 90.3%, respectively. In addition to this, the simulated study shows an accuracy of 78.5%, signifying that the proposed method performs exceptionally. To our knowledge, this is the first application of supervised machine learning methods to be used to correct misclassification in FFQ data that does not require calibration data to be collected.

Machine learning (ML) methods have been used to optimize prediction of FFQ data with methods such as dimensionality reduction; however, it has not been utilized previously in the correction of measurement error [22]. Hence, in this paper, we explore the use of ML to adjust measurement error. Several machine learning models such as decision trees and multinomial logistic regression were considered for use as the classification model in this analysis. However, accuracy and model simplicity were chosen to be the most important characteristics for a good model; therefore, random forest proved to be the best performer. We have reflected this on our simulated dataset in the table below (Table 1). Random forest works as an aggregate of multiple random decision trees, which gives an accuracy advantage over other methods [21]. In addition, it is possible to rank the most important variables influencing the responses [21].

**Table 1:** Table showing a comparative analysis of accuracy, precision, recall and F1-Score for three distinct models on simulated dataset: multinomial logistic regression, decision trees and random forest

	**Multinomial Logistic Regression**	**Decision Trees**	**Random Forest**
**Accuracy**	70.00%	59.05%	78.50%
**Precision**	0.715	0.600	0.794
**Recall**	0.700	0.590	0.786
**F1-Score**	0.701	0.592	0.785

Previous studies have shown the use of other methods such as regression calibration and generalized gamma regression to adjust for measurement error [8]. These methods use a generalized linear model to show diet-disease association, and directly model bias in them. Knowing that true intake is generally measured incorrectly or is missing, these methods express the newly corrected data points as the conditional expected value of the unobserved true intake, given the observed data with error and error-free covariates [8]. These new data points are then used as the observations for the diet-disease model and replace the previous observations. However, a lot of parameters, steps and instruments are involved in this process, hence contributing to the additional noise in the model. One of the instruments used, a 24HR recall has a distribution that is characterized by skewness due to excess zeros in the dataset. It is also characterized by heteroskedasticity, meaning higher variability than the FFQ dataset [23]. The regression calibration method also involves a Box-cox transformation to normalize the 24HR recall data, and an inverse transformation to bring it back to its original scale. This means that between-person correlations would be lost. Generalized gamma regression combats this as the true intake is modeled as the product of the conditional mean and mean probability of the gamma distribution of the individual variables [8].

Our proposed method has many advantages over regression calibration methods. It does not require 24HR as an additional tool. It relies on the derived correlations and the underlying ground truth in the FFQ dataset, hence ensuring no unnecessary introduction of variability in the data or participant burden. This works because of low-rank assumption. It also does not involve a transformation of the variables or the introduction of any other distribution. This is because the subset of data containing the underlying truth in the FFQ dataset assumes normality. Our method considers the measurement error in the FFQ data as an aggregate of the measurement error in multiple covariates in the data and adequately adjusts the error concurrently. Another significant difference is that current methods are fully parametric, hence, inefficient. Our proposed method involves fewer parameters and is more computationally efficient. Finally, we see high accuracy measures for the models used, hence showing the efficiency of our proposed method.

There are some limitations to be considered. Previous research uses energy intake calibration with known biomarkers, such as doubly labeled water or urinalysis, to determine true energy intake. However, FFQs are not designed to quantitatively estimate total energy intake, due to the finite list of food and beverages, and limited data on food specificity. In addition to this, though we have successfully derived a method to tackle incorrect observations caused by underestimations, future research should address the other FFQ measurement challenges which are overreported observations and missing data points. Knowing that food frequency questionnaires query a finite set of foods and beverages [19], it is fair to assume that certain foods will be omitted. This increases the issue of under-reporting; however, there are instances where over-reporting happens (e.g., vegetable consumption). These analyses will be done in further studies.

## Conclusion and future work

This research presents an alternative and novel method to reduce the measurement error in FFQ datasets using the RF classifier model and an additional underreported data adjustment algorithm to recover the “true” predicted classes. This method efficiently reduces misclassification due to underestimation in self-reported dietary data estimated by FFQ.

In future work, the ML techniques to adjust for the missing entries in the dataset and overreporting will be explored further. These have also contributed to the challenges faced by current researchers using the FFQ dataset. We will also consider the use of deep learning methods to accurately combat the missing data challenges and mitigate measurement error in the datasets. Machine learning has proven to be an invaluable tool for error adjustment and could be useful to address numerous measurement error problems.

### Supplementary Information

**Additional file 1: Table A.1.** Body fat norms by age and sex for women. **Table A.2.** Body fat norms by age and sex for men.

## Data Availability

The data that support the findings of this study are available from the Emory University Predictive Health Institute and can be accessed via application at https://predictivehealth.emory.edu/research/resources.html.

## Abbreviations

FFQ: Food Frequency Questionnaire
RF: Random Forest
24HR: 24 Hour Recalls
FR: Food Record
REGARDS: Reasons for Geographic and Racial Differences in Stroke
ARIC: Atherosclerosis Risk in Communities Study
ML: Machine Learning
CHWDB: Center for Health Discovery and Well Being Database
F: Frequency
Q: Quantity
LDL: Low-density Lipoprotein
BMI: Body Mass Index
