# Supplementary material for: Mitigating underreported error in food frequency questionnaire data using a supervised machine learning method and error adjustment algorithm
Source: BMC Med Inform Decis Mak. 2023 Sep 9;23:178. doi: 10.1186/s12911-023-02262-9 (PMC10492312; doi:10.1186/s12911-023-02262-9)
Supplement: Supplementary file 1 — Additional file 1: Table A.1. Body fat norms by age and sex for women. Table A.2. Body fat norms by age and sex for men. [file 12911_2023_2262_MOESM1_ESM.docx]

**Appendix A**

**Table A**

This table shows the classifications of body composition in relative age groups, according to the PennRec’s Body composition Information and FAQ’s sheet [20]:

**Table A.1.** Body fat norms by age and sex for women

| **%Body Fat/Age** |  |  |  |  |  |
| --- | --- | --- | --- | --- | --- |
|  | **20-29** | **30-39** | **40-49** | **50-59** | **60-69** |
| Low (increased health risk) | < 14 | < 14 | < 14 | < 14 | < 14 |
| Excellent/ Fit (Healthy) | </= 16.5 | </= 17.4 | </= 19.8 | </= 22.5 | </= 23.2 |
| Good/Normal (Healthy) | 16.6-19.4 | 17.5-20.8 | 19.9-23.8 | 22.6-27 | 23.3-27.9 |
| Fair/ Average (Healthy) | 19.5-22.7 | 20.9-24.6 | 23.9-27.6 | 27.1-30.4 | 28-31.3 |
| Poor (increased health risk) | 22.8-27.1 | 24.7-29.1 | 27.7-31.9 | 30.5-34.5 | 31.4-35.4 |
| High (Increased health risk) | > 27.2 | > 29.2 | > 31.3 | > 34.6 | > 35.5 |

**Table A.2.** Body fat norms by age and sex for men

| **%Body Fat/Age** |  |  |  |  |  |
| --- | --- | --- | --- | --- | --- |
|  | **20-29** | **30-39** | **40-49** | **50-59** | **60-69** |
| Low (increased health risk) | < 8 | < 8 | < 8 | < 8 | < 8 |
| Excellent/ Fit (Healthy) | </= 10.5 | </= 14.5 | </= 17.4 | </= 19.1 | </= 19.7 |
| Good/Normal (Healthy) | 10.6-14.8 | 14.6-18.2 | 17.5-20.6 | 19.2-22.1 | 19.8-22.6 |
| Fair/ Average (Healthy) | 14.9-18.6 | 18.3-21.3 | 20.7-23.4 | 22.2-24.6 | 22.7-25.2 |
| Poor (increased health risk) | 18.7-23.1 | 21.4-24.9 | 23.5-26.6 | 24.7-27.8 | 25.3-28.4 |
| High (Increased health risk) | >/= 23.2 | >/= 25 | >/= 26.7 | >/= 27.9 | >/= 28.5 |
